# Development of a new pre-vascularized tissue-engineered construct using pre-differentiated rADSCs, arteriovenous vascular bundle and porous nano-hydroxyapatide-polyamide 66 scaffold

**DOI:** 10.1186/1471-2474-14-318

**Published:** 2013-11-08

**Authors:** Pei Yang, Xin Huang, Jacson Shen, Chunsheng Wang, Xiaoqian Dang, Henry Mankin, Zhenfeng Duan, Kunzheng Wang

**Affiliations:** 1 Department of Orthopaedics, Second Affiliated Hospital of Medical College of Xi’an Jiaotong University, No. 157 Xiwu Road, 710004 Xi’an, Shaanxi, China; 2 Department of Cardiology, First Affiliated Hospital of Medical College of Xi’an Jiaotong University, Ion Channel Disease Laboratory, Key Laboratory of Environment and Genes related to Diseases of Education Ministry, 710061 Xi’an, Shaanxi, P.R.China; 3 Department of Biological Chemistry, Wellesley College, 02481 Wellesley, Massachusetts, USA; 4 Department of Orthopaedic Surgery, Massachusetts General Hospital and Harvard Medical School, Boston, Massachusetts, USA; 5 Sarcoma Biology Laboratory, Center for Sarcoma and Connective Tissue Oncology, Massachusetts General Hospital and Harvard Medical School, Boston, Massachusetts, USA

**Keywords:** Adipose-derived stem cells, Tissue engineering, Angiogenesis, Scaffolds, Prefabrication

## Abstract

**Background:**

Development of a pre-vascularized tissue-engineered construct with intrinsic vascular system for cell growth and tissue formation still faces many difficulties due to the complexity of the vascular network of natural bone tissue. The present study was to design and form a new vascularized tissue-engineered construct using pre-differentiated rADSCs, arteriovenous vascular bundle and porous nHA-PA 66 scaffold.

**Methods:**

rADSCs were pre-differentiated to endothelial cells (rADSCs-Endo) and then incorporated in nHA-PA 66 scaffolds *in vitro*. Subsequently, *in vivo* experiments were carried out according to the following groups: Group A (rADSCs-Endo/nHA-PA 66 scaffold with arteriovenous vascular bundle), Group B (rADSCs/nHA-PA 66 scaffold with arteriovenous vascular bundle); Group C (nHA-PA66 scaffold with arteriovenous vascular bundle), Group D (nHA-PA 66 scaffold only). The vessel density and vessel diameter were measured based on histological and immunohistochemical evaluation, furthermore, the VEGF-C, FGF-2 and BMP-2 protein expressions were also evaluated by western blot analysis.

**Results:**

The results of *in vivo* experiments showed that the vessel density and vessel diameter in group A were significantly higher than the other three groups. Between Group B and C, no statistical difference was observed at each time point. In accordance with the results, there were dramatically higher expressions of VEGF-C and FGF-2 protein in Group A than that of Group B, C and D at 2 or 4 weeks. Statistical differences were not observed in VEGF-C and FGF-2 expression between Group B and C. BMP-2 was not expressed in any group at each time point.

**Conclusions:**

Compared with muscular wrapping method, arteriovenous vascular bundle implantation could promote vascularization of the scaffold; and the angiogenesis of the scaffold was significantly accelerated when pre-differentiated rADSCs (endothelial differentiation) were added. These positive results implicate the combination of pre-differentiated rADSCs (endothelial differentiation) and arteriovenous vascular bundle may achieve rapidly angiogenesis of biomaterial scaffold.

## Background

Clinically, autologous bone grafting with arteriovenous vascular bundle implantation has been widely used for the treatment of avascular necrosis of the femoral head [1], bone defects [2] and non-union [3,4]. Though the technique is viewed as the “gold standard” [5] for its therapeutic safety and efficacy, many complications may arise such as wound issues, vessel injuries and bleeding which require a secondary surgical operation [6,7]. This leads us to propose the development of a pre-vascularized construct that would provide an intrinsic vascular system for cell growth and tissue development.

Undoubtedly, tissue-engineered bone and cartilage can be successful constructed both *in vitro* and *in vivo*[8,9]. However, the technique of forming an intrinsic vascular system within the bone tissue-engineered scaffolds remains a challenge due to the complexity of the vascular network of natural bone tissue [10]. Several extensive studies have been carried out to accelerate the vascularization process in bone tissue engineering [11]. Compared with other methods, the application of non-functional pre-existing blood vessels *in vivo* as a vascular carrier and incorporation of biomaterials and cells or growth factors into them is advantageous, as it allows for instantaneous perfusion after the graft is implanted, which can dramatically decrease the time required for capillary ingrowth [10,12-15]. Arteriovenous vascular loop (AV-loop) [10,13,16] and arteriovenous vascular bundle (AV-bundle) [10,15,17] are recognized as pre-existing blood vessels, which have been used in animal experiments. Furthermore, AV-bundle has been used for clinical treatment [1-4]. Theoretically, the potential mechanisms of accelerated angiogenesis by the AV-loop and AV-bundle have been proposed as follows [18]: (1). Inflammatory responses caused by surgical trauma promote the releasing of inflammatory factors, which physiologically increase vascular permeability, and promoted capillary network building; (2). Local matrix hypoxic conditions lead to the up-regulation of hypoxia inducible factor (HIF-1) expression and subsequently up-regulate the expression of angiogenic factors such as vascular endothelial growth factor (VEGF), which results in cascade amplification to increase vascular permeability and to stimulate the proliferation of endothelial cells and maintain the physiological function of its differentiated state; (3). Vascular flow shear stress (FSS) played an important role in adult angiogenesis process. High FSS could promote the growth of collateral vessels whose growth has stopped, and the number of microvessels has increased significantly [12,18,19].

Compared with the direct use of angiogenic factors in the pre-vascularized procedures, the application of angiogenic cells may provide a suitable method of continuous local delivery of angiogenic cytokines through autocrine/paracrine mechanism for extended periods [16]. Endothelial progenitor cells (EPCs) [20] and human umbilical vein endothelial cells (HUVECs) [21] have been previously transplanted into biomaterial scaffolds, demonstrating that the cells can accelerate angiogenesis. However, limited sources will hamper their clinical application. Mesenchymal stem cells isolated from adipose tissue (ADSCs) demonstrate similar multilineage differentiation potencies (including endothelial differentiation) with bone-marrow derived mesenchymal stem cells (BMSCs), which are widely investigated in bone tissue engineering [22]. The use of ADSCs rather than BMSCs may be advantageous in that greater cell numbers can be harvested from the patient with less pain. As well, ADSCs are reported to have positive effects on patients who received bone marrow transplantation and suffered from GVHD (graft versus host disease), suggesting that they have an immunomodulatory function [23]. These results suggest that ADSCs may be an attractive cell candidate for the prefabrication of vascularized construct.As for the biomaterials scaffold, the shape of the scaffold must be controlled and customized. Three-dimensional scaffolds made of biomaterials such as nano-hydroxyapatite-polyamide 66 (nHA-PA 66) have been shown to be an effective composition material candidate for three-dimensional scaffolds due to its favorable biocompatibility/chemical composition osteoconductivity and bioactivity [24-26].

In the present study, rat ADSCs (rADSCs) were pre-differentiated to endothelial cells, and then incorporated in nHA-PA 66 scaffolds *in vitro*. Subsequently, the composites were implanted with or without AV-bundle *in vivo*. We hypothesized that rADSCs derived endothelial cells together with AV-bundle would accelerate vascularity of the scaffolds *in vivo*.

## Methods

### ***In vitro experiments***

#### ***The characteristic of the nHA-PA 66 scaffold***

The nHA-PA 66 scaffold was synthesized from nano-hydroxyapatite and polyamide 66 foamed by the thermal pressing and the injection molding techniques by Sichuan Guona Technology Co., Ltd (Chengdu, Sichuan, China). The biomechanical properties (including elastic modulus, bending strength and compressive strength) and porosity were tested according to the methods reported previously [27] (*n* = 6, respectively). Another six nHA-PA 66 scaffolds were used for ultrastructure evaluation based on scanning electron microscopy (SEM) to observe the micro-architecture. To adapt to AV-bundle embedding *in vivo*, a side groove was made that passed through the scaffold along its long axis.

#### ***rADSCs isolation and cultivation***

This study was carried out in strict accordance with the recommendations in the *Guide for the Care and Use of Laboratory Animals of the National Institutes of Health*. The protocol was approved by the *Committee on the Ethics of Animal Experiments of the Xi’an Jiaotong University*.

An aseptic cut of Sprague Dawley (SD) rat adipose tissue was performed, and adipose-derived stem cells were extracted in accordance with the conventional method [28]. The third passage of rADSCs (P3 rADSCs) were obtained for evaluating the multilineage differentiation capacity after flow cytometry confirmation.

#### ***Flow cytometry***

Briefly, P3 rADSCs were trypsinized and incubated with fluorescein conjugated antibody against CD29, CD34, CD44 and CD45 (Santa Cruz, CA, USA) at 4°C in 0.5% BSA and 2 mM EDTA in PBS for 30 min. Subsequently, the labeled cells were run on a BD FACSCanto II flow cytometer (BD, CA, USA) to identify the phenotypes.

#### ***Osteogenic induction and adipogenic induction***

Osteogenic induction experiments were conducted using previous methods with minor modifications [29]. At week 2 of culture, alkaline phosphatase (ALP) calcium cobalt staining was conducted, and at week 4 of culture, alizarin red staining was conducted. Adipogenic induction experiments were also carried out according to the methods previously described [29], and Oil Red O staining was used to confirm the inductive efficiency.

#### ***Endothelial differentiation and confirmation***

P3 rADSCs were suspended in endothelial differentiation medium (medium 199 + 50 ng/ml VEGF + 10 ng/ml b-FGF + 3% FBS) at a density of 1 × 10^5^/ml and 0.5 ml of cell suspension was added to each well of a 12-well plate. Cultures were incubated at 37°C in a 5% CO_2_. The medium was changed 3 times for 8 days. Differentiation was confirmed by angiogenesis assay and immunocytochemistry.

#### ***Angiogenesis assay***

After rADSCs were differentiated with endothelial differentiation medium for 8 days, the cells were trypsinized and seeded a 24-well plate which was coated with Matrigel (8.8 mg/ml; BD,USA) at a concentration of 5 × 10^4^/well in endothelial differentiation medium. Cultures were incubated at 37°C in a 5% CO_2_ humidified atmosphere for 48 h and observed with an inverted photomicroscope.

#### ***Western blot analysis for von willebrand factor expression***

After rADSCs were differentiated with endothelial differentiation medium for 8 days, the cells were trypsinized and the proteins were also prepared for western blot assay of von Willebrand factor [(rabbit anti-rat vWF polycolonal antibody, Santa Cruz, CA, USA)] as described previously [30]. Mouse anti-rat β-actin (Sigma, MO, USA) antibody was used as internal control gene. The un-differentiated P3 rADSCs were used as control group.

#### ***In vitro construction of rADSCs-Endo/nHA-PA 66 scaffold composites and rADSCs/nHA-PA 66 scaffold composites***

After rADSCs were differentiated with endothelial differentiation medium for 8 days (termed as rADSC-Endo), the density of the rADSCs-Endo cells were adjusted to 2 × 10^4^/mL and the cells were seeded into the scaffolds with 1 ml in each (termed as rADSCs-Endo/nHA-PA 66 scaffold composite, *n* = 28). Two rADSCs-Endo/nHA-PA 66 scaffold composites were removed respectively at 3 and 7d during co-culturing, and after conventional treatment, SEM was use to evaluate the composite structure of cells and scaffolds. The composites prepared using the same methods with a substitution of P3 rADSCs for rADSCs-Endo cells were termed rADSCs/nHA-PA 66 scaffold composites (*n* = 24).

### In vivo experiments

#### ***Animals and study groups***

96 SD rats (male, weighing 350–450 g) were used and assigned randomly into 4 groups according to the different composites used. Prior to experimentation, all rats were housed in a temperature-controlled room under a 12 hr/12 hr-light/dark and were allowed access to standard rat chow and tap water *ad libitum*. All surgical procedures were conducted under aseptic conditions and general anesthesia (pentobarbital, 30 mg/kg).

#### ***Surgical procedures***

**Figure 1:**
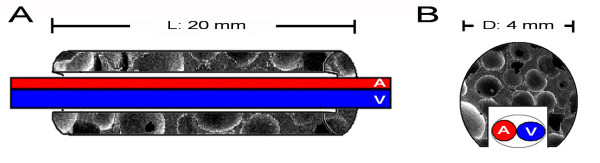
**Schematic diagrams (A: longitudinal view; B: trasversial view) demonstrate the relationship between C-shape nHA/PA66 scaffold and the implanted AV-bundle.** A: implanted artery; V: implanted vein.

Through a 2 cm skin incision parallel to the left inguinal ligament, the soft tissues around the inferior epigastric artery and vein were carefully removed, and the AV-bundle was fully exposed. For Group A (*n* = 24), the AV-bundle was inserted into the side groove of the rADSCs-Endo/nHA-PA 66 scaffold composite and fixed with surrounding tissue. A schematic outline of the surgical procedures was shown in Figure 1. For Group B and Group C (*n* = 24, respectively), the AV-bundle was inserted into the side groove of the rADSCs/nHA-PA 66 scaffold composite and nHA-PA 66 scaffold respectively. For Group D (*n* = 24), the nHA-/PA 66 scaffold was directly embedded into quadriceps without AV-bundle.

Incisions were then closed with a 1–0 fiber thread suture line in a routine fashion. The animals were monitored post-operatively. At 2 and 4 weeks after surgery, twelve rats from each group were sacrificed under general anesthesia (*n* = 6 for histological evaluation and western blot assay, respectively). Blood vessels were broken and the implants were removed in Group A, B and C. All the samples were fixed with 4% paraformaldehyde for 24 h. After full decalcification with 20% EDTA, histological and immunohistochemical staining was conducted.

#### ***Histological and immunohistochemical evaluation***

At 2 and 4 weeks postoperatively (*n* = 6 for each group), the implants with the surrounding tissues were retrieved. The samples were cut into 8-μm sections and stained with Masson-trichrome staining for histological evaluation.

The conventional method was employed for vWF immunohistochemical staining [31]. The degree of scaffold vascularization was observed under upright microscope in 200× or 400× amplification field.

#### ***Histological quantitative analysis***

A light microscope (Leica, Germany) was used for histological evaluation. 6 transverse serial sections in the central parts of the scaffolds were used for histomorphometrical evaluation using computer-based image analysis techniques (Leica Qwin Pro-image analysis system, Germany). The following parameters were determined by digital analysis in a blinded manner.

1. Vessel density [32]. For Masson trichrome stained sections, structures were identified as vessels if they met two of the three following criteria: the presence of an endothelial cell lining, a well-defined lumen and the presence of red blood cells. In sections labeled with vWF, the structures that were stained brown and had a well-defined lumen were counted as blood vessels. The number of vessels in the section was counted manually at 200× magnification, and the vessel density was represent as the number of vessels/mm^2^.

2. Vessel diameter [32]. For each vessel, the least diameter, i.e. the two diametrically opposed points on the luminal microvessel wall, was identified at 400× magnification.

In vivo VEGF-C, fibroblast growth factor 2 (FGF-2) and bone morphogenetic protein 2 (BMP-2) protein expression detection by western blot analysis

After retrieval from the rats, the implants (*n* = 6 for each group at 2 and 4 weeks, respectively) were extensively washed with PBS and placed in a pre-cooled mortar and was ground within the liquid nitrogen for protein extraction. Total 50 μg proteins were loaded for electrophoresis on SDS-polyacrylamide gel, and then transferred to PVDF membranes. Rabbit anti-rat VEGF-C polyclonal antibody, rabbit anti-rat FGF-2 polyclonal antibody and rabbit anti-rat BMP-2 polyclonal antibody (Santa Cruz, CA, USA) were diluted at a concentration of 1:500, 1:200 and 1:100 respectively. The working concentration of internal control mouse anti-rat β-actin monoclonal antibody (Sigma, MO, USA) was 1:2000. The antibodies were incubated at 4°C for overnight. The membranes were then incubated with horseradish peroxidase labeled anti-rabbit IgG (for detection of VEGF-C, FGF-2 and BMP-2) and anti-mouse IgG (for detection of β-actin) with the dilution of 1:500 at room temperature for 2 h. The protein bands were visualized by DAB staining. The ratio of the intensities of the target genes and β-actin bands was used to represent the level of the target gene protein expression.

### Statistical analysis

SPSS11.0 statistical software was used for analysis. The data were expressed as mean ± standard deviation. The analysis of variance (ANOVA) was used for group comparison, and *post hoc* test was used for pairwise comparison (inspection level α = 0.05).

## Results

### ***In Vitro experiments***

#### ***The characteristic of the nHA-PA 66 scaffold***

**Figure 2:**
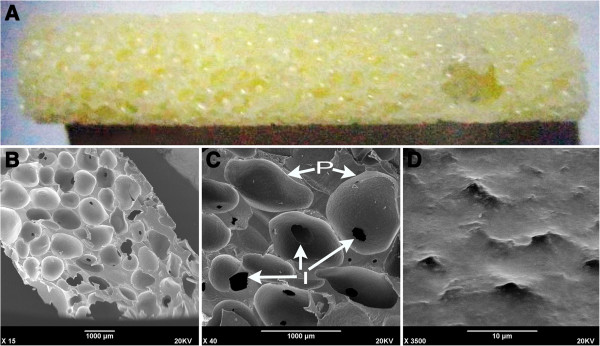
**Gross view (A) and SEM photomicrograph of the nHA-PA66 scaffold (B, C and D). B**, **C**: Lower magnification of the surface of the scaffold. **D**: Higher magnification showed the wall of the macropores. P, pore; I, interconnecting path.

The biomechanical property including elastic modulus, bending strength and compressive strength were shown in Table 1, which were similar to those of the natural bone [33]. Under gross view, the scaffold exhibited a cylindrical type with the diameter of bottom surface as 4.0 mm and the height as 20 mm (Figure 2A). It was found that the material exhibited a porous surface, and there were interconnections between macropores. Under higher magnification, macropore exhibited smooth walls (Figure 2B-2D). The porosity was (68.41 ± 9.20) %, macropore size was (620.16 ± 111.85) μm and interconnection pore size was (185.41 ± 84.25) μm; these parameters were in accordance with previously reported [26]. To adapt to vascular bundle embedding, a side groove (width: 2.0 mm) which passed through the scaffold along its long axis was made in each of the scaffolds.

**Table 1:** Physical properties of the porous nHA/PA66 scaffold

**Porosity ( *% *)**	**Macropore diameter ( *μm *)**	**Interconnection diameter ( *μm *)**	**Elastic modulus ( *Gpa *)**	**Bending strength ( *Mpa *)**	**Compressive strength ( *Mpa *)**
68.41 ± 9.20	620.16 ± 111.85	185.41 ± 84.25	6.25 ± 0.82	85.14 ± 12.13	100.12 ± 18.95
			3-25*	90-95*	110-125*

*indicate to nature bone.

#### ***rADSCs morphological observation, osteogenic and adipogenic induction observation***

**Figure 3:**
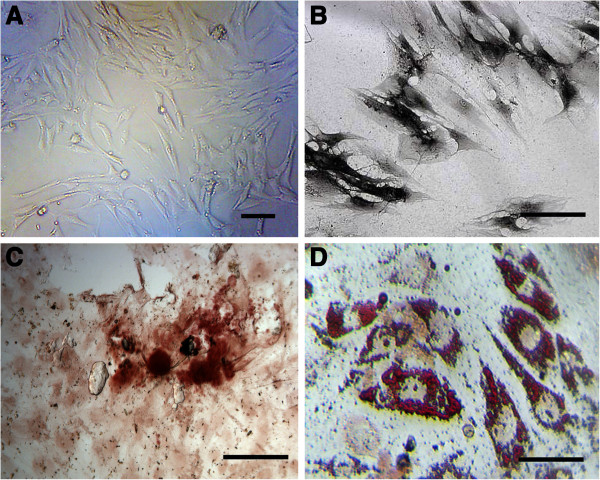
**Examination of rADSCs differentiation capacity into osteogenic and adipogenic lineages. A**: P3 rADSCs. **B** and **C**: cells were positive for alkaline phosphatase staining and alizarin red staining after osteogenic induction. **D**: cells were positive for oil red staining after adipogenic induction, indicating they differentiated into mature adipocytes. Bars indicate 100 μm.

rADSCs exhibited the morphology of fibroblastoid mononuclear cells (Figure 3A). After osteogenic induction, ALP activity and mineralized matrix deposition were confirmed by ALP staining (Figure 3B) and alizarin red staining (Figure 3C). Oil Red O staining after adipogenic induction was performed to detect lipid accumulation. Many orange-red lipid droplets of different sizes were seen in the cytoplasm; additionally, there were droplets that accounted for 80% to 90% of the entire cell volume (Figure 3D).

#### ***Flow cytometry***

**Figure 4:**
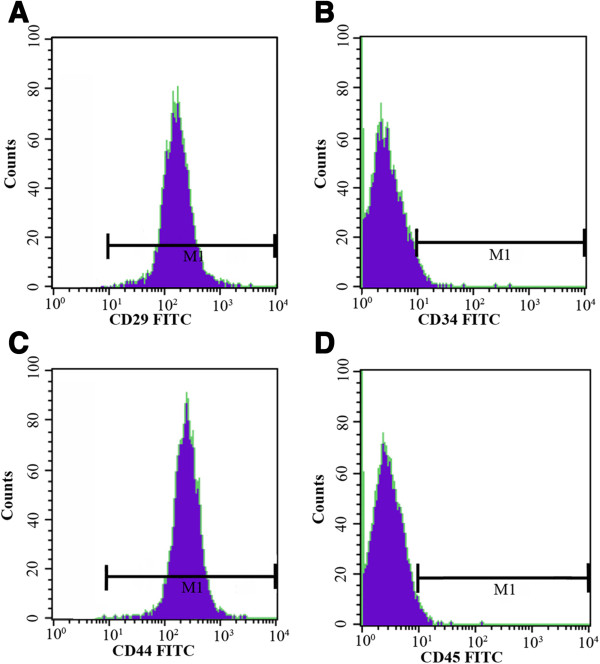
**Flow cytometry analysis of rADSCs.** P3 rADSCs are CD34 and CD45 negative **(B and D)**, but CD29 and CD44 positive **(A and C)**.

Flow cytometry demonstrated that the cultured P3 rADSCs were positive for CD29 and CD44 but negative for CD34 and CD45 (Figure 4A-D). The phenotypes were in accordance with those reported by Xu YF *et al.*[34].

#### ***Endothelial differentiation and confirmation***

**Figure 5:**
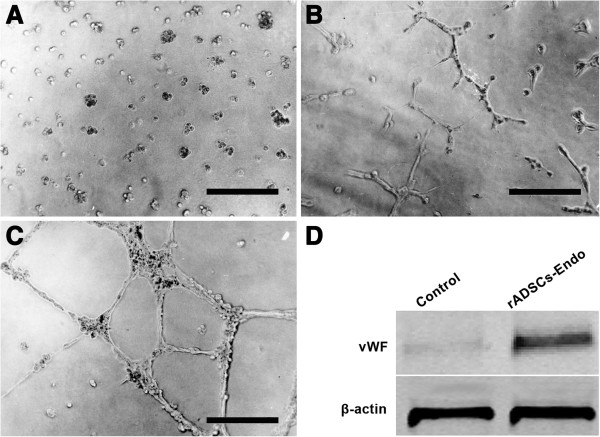
**Angiogenesis assay.** After rADSCs were differentiated in endothelial differentiation medium for 8 days, the differentiated cells were placed in Matrigel in a 24-well plate. **A**: 1 hour after cell-seeding; **B**: 24 hours after seeding, exhibit partial tubule formation, **C**: 48 hours after cell seeding, clearly demonstrated capillary-like networks between cells. **D**: Western blot analysis using anti-vWF revealed up-regulation of vWF in rADSCs-Endo group. Bars indicate 100 μm.

After rADSCs were differentiated with endothelial differentiation medium for 8 days, the cells were trypsinized and seeded in a 24-well plate coated with Matrigel for angiogenesis assay (Figure 5A). During the first 24 h, cells spread randomly, moved, and started to form small and seldom interconnected clusters (Figure 5B). At 48 h, clusters increased in size and were highly connected, discrete Matrigel areas were empty and surrounded by cell islets or chains (Figure 5C). Based on western blot analysis, the protein expression of vWF was also detected after rADSCs were differentiated with endothelial differentiation medium for 8 days (Figure 5D).

#### ***In vitro construction and testing of rADSCs-Endo/nHA-PA 66 scaffold composites***

**Figure 6:**
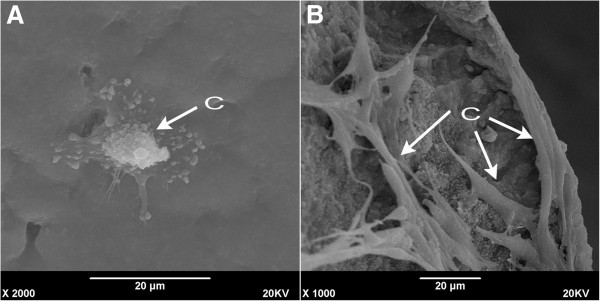
**SEM photomicrograph of rADSC/nHA-PA 66 scaffold composite. A**: At 3 days after seeding. **B**: At 7 days after cell seeding. C, rADSCs-Endo cells.

At 3d after co-culturing of rADSCs-Endo cells and the scaffolds, the number of the cells in the scaffolds reduced, while cell morphology was not fully extended with a small amount of matrix secretion (Figure 6A); At 7d, the number of the cells significantly increased, and the morphology was fully extended and long fusiform (Figure 6B).

### ***In vivo experiments***

#### ***Clinical and physical examinations***

95 of 96 rats survived over the time course of the study; one rat of group B was died during anesthesia, and severe infection was noted in one rat in group B. Therefore, two additional rats were operated on to maintain the experimental design numbers (total number of rats, 98).

#### ***Histological and immunohistochemical evaluation***

At 2 and 4 weeks after surgery, the Masson’s trichromestained sections from each group showed that the scaffolds were in-grown together with fibrous connective tissues and blood vessels. In group A, B and C, when the scaffolds were retrieved at both 2 and 4 weeks, noticeable bleeding occurred due to the implanted AV-bundle, which indicated the vessels did not blockage by the thrombosis *in vivo.* In group D, the samples were encapsulated with fibrous tissue.

**Figure 7:**
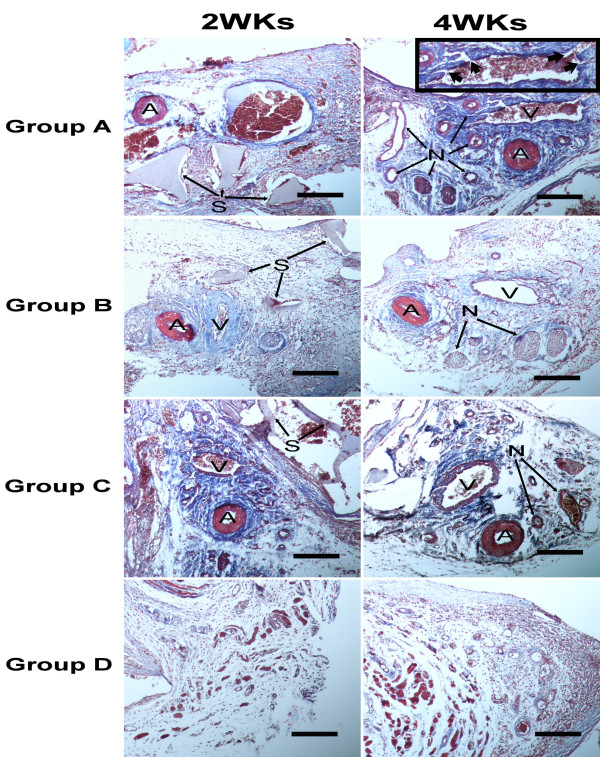
**Histological observation of the scaffolds of group A, B, C and D at 2 and 4 weeks after implantation.** Masson trichrome stain. A: implanted artery; V: implanted vein; N: neovessels; S: residual scaffold. Bold arrow indicates the luminal sprouting from the vein. Bars indicate 500 μm.

**Figure 8:**
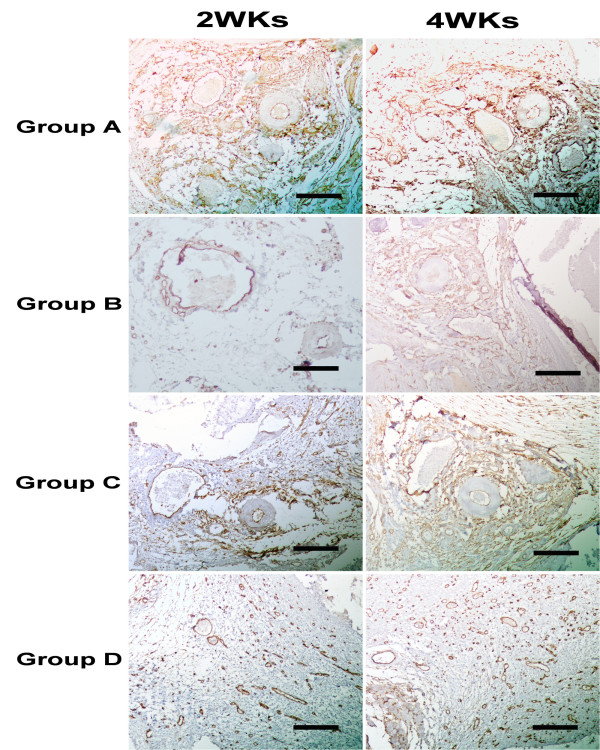
**Typical immunohistochemical images for vessels of vWF antibody of group A, B, C and D at 2 and 4 weeks after implantation (brown staining).** Bars indicate 500 μm.

Histologically, at 2 weeks after surgery in Group A, B and C, newly formed vessels were prominent in the AV-bundle and the adjacent tissue, but the diameter of newly formed vessels was small. At 4 weeks in Group A the number of newly formed vessels significantly increased around the implanted AV-bundle, and the diameter was larger. Small arteries were also observed in Group A but not in Group B and C. While only some immature capillaries were observed in Group D (Figures 7 and 8). Generally, luminal sprouting from the inferior epigastric vein was observed in group A at 4 weeks. In all groups, osteoid and osteoblast were not observed both at 2 or 4 weeks after surgery.

#### ***Histological quantitative analysis***

**Figure 9:**
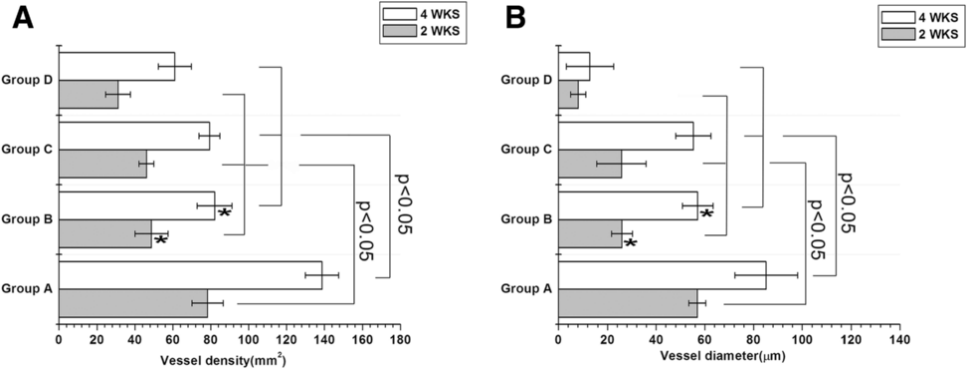
**Vessel density (A), vessel diameter (B) analysis of each group at 2 and 4 weeks after implantation.** * indicates compared with Group C at the same time point, *p* > 0.05.

At 2 weeks after surgery, the vessel density in Group A (78.31 ± 8.25)/mm^2^ was significantly higher than Group B and C [(48.72 ± 8.73)/mm^2^ and (46.03 ± 3.97)/mm^2^] (both *p<0.05*). Both Group A, B and C were significantly higher than group D (31.04 ± 6.54)/mm^2^ (*p<0.05*). At 4 weeks, the vessel density in Group A (138.74 ± 8.82)/mm^2^ were also higher than that of Group B and C [(82.02 ± 9.17)/mm^2^ and (79.28 ± 5.57)/mm^2^] (both *p<0.05*). Again, Group A, B and C were also significantly higher than group D (61.02 ± 8.74)/mm^2^ (both *p<0.05*). Significant difference was also presented in group A at 2 weeks vs Group A at 4 weeks (*p<0.05*), the same trends were also observed in group B, C and D. Between Group B and C, no statistical differences were observed at each time point for vessel density (Figure 9A).

The vessel diameter at 2 weeks in Group A [(56.87 ± 3.45) μm] was larger than that in Group B [(25.94 ± 4.27) μm], Group C [(25.71 ± 10.12) μm] and Group D [(8.12 ± 3.08 μm] (both *p<0.05*). At 4 week after surgery, the vessel diameter in Group A [(85.20 ± 12.88) μm] was still larger than that in Group B [(57.02 ± 6.30) μm], Group C [(55.28 ± 7.25) μm] and Group D [(12.87 ± 9.68) μm] (both *p<0.05*). Significant differences were also observed within all of the groups at the two time points. Reflective of vessel density, no statistical differences were observed between Group B and C at each time point for vessel diameter (Figure 9B).

#### ***VEGF-C, FGF-2 and BMP-2 protein expression analysis***

**Figure 10:**
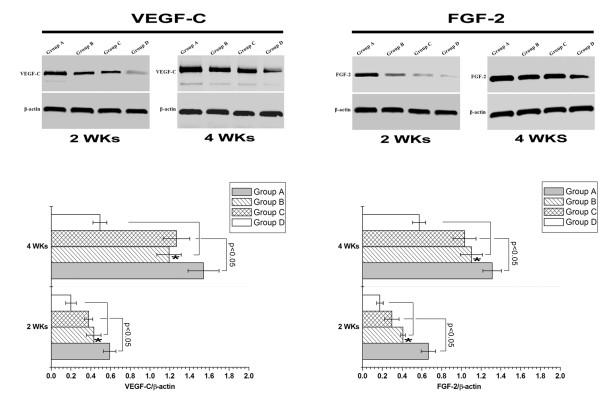
**Western blot results of VEGF-C and FGF-2 of each group at 2 and 4 weeks demonstrated increased protein expression in Group A.** * indicates compared with Group C at the same time point, *p* > 0.05.

We further confirmed the expression pattern of VEGF-C, FGF-2 and BMP-2 *in vivo* by western blot in each group at different time points post-surgery. As demonstrated in Figure 10, there was dramatically higher expression of VEGF-C and FGF-2 protein in Group A than the other three groups at 2 or 4 weeks (both *p<0.05*). Statistical differences were observed on the expression of VEGF-C and FGF-2 protein at each time point between Group B and D or Group C and D (both *p<0.05*). Statistical differences were not observed in VEGF-C and FGF-2 expression between Group B and C in each time point (both *p*<0.05). BMP-2 was not expressed in each group at each time point (Additional file 1: Figure S1).

## Discussion

In the present study, we successfully constructed a novel tissue engineered construct by using pre-differentiated rADSCs (endothelial differentiation) and porous nHA-PA 66 scaffold *in vitro*. Subsequently, the inferior epigastric AV-bundle was directly clipped in the composite for evaluating its capability of angiogenesis *in vivo*. The results revealed that compared with muscular wrapping method, AV-bundle implantation could promote vascularization of the scaffold; and the angiogenesis of the scaffold was significantly accelerated when pre-differentiated rADSCs (endothelial differentiation) were added. These positive results implicate the combination of pre-differentiated rADSCs (endothelial differentiation) and AV-bundle may achieve rapidly angiogenesis for biomaterial scaffold.

Tissue-engineered bone without intrinsic vascular network is recognized as “dead bone”. The process of revascularization *in vivo* usually requires a long period of time, and implanted bone necrosis and trauma non-union may occur, which is suggested to be caused by cell death of area farther from the capillaries [35]. Generally, the distribution of cells is limited to a distance of 150–200 μm away from the nearest capillary which is the effective diffusion range of nutrients and oxygen [36,37]. Therefore, assembly of a vascular network with the necessary vessel to exchange oxygen and nutrients to cells within the scaffold is crucial for the survival of cells and healing efficacy of the tissue engineered graft after implantation *in vivo*.

In 1993, Mikos et al. initiated the concept of pre-vascularized tissue engineered grafts based on the different performance of pre-vascularized and non-vascularized scaffolds *in vivo*[38]. The vascular system inside the bone tissue not only enhances the survival of bone tissue cells, but also produces growth cytokines and mesenchymal stem cells to promote bone metabolism and repair. Furthermore, abundant blood supply may prevent infection [10,13-15,36-38]. Many researches have been focused on prefabrication of vascular network *in vitro*. While after the *in vitro* prefabricated graft with capillary network has been implanted *in vivo*, the vascular anastomosis between the host vessels and the prefabricated capillaries could not be achieved within a short period due to the limitation of host vascular ingrowth rate of several tenths of micrometers per day [39].

Recent studies have demonstrated that axial vascularization in engineered grafts could be prefabricated *in vivo* using the pre-existing vessels, thus accelerating the repair of bone injury, so called “*in vivo* bioreactor” technique [10-17]. As for the pre-existing vessels, several studies confirmed that AV-loop could effectively accelerate the angiogenesis process of the scaffold *in vivo*[13,16]. For this technique, femoral artery-vein and saphenous artery-vein models were the most widely used to construct the AV-loop. Unfortunately, the technique destroys the blood supply at donor site, which is needed for adequate progression of arteriovenous anastomosis. AV-bundle is another choice to prefabricate the vascularized graft [15,17,40]. Some scholars have used thoracodorsal AV-bundle, femoral AV-bundle, deep femoral AV-bundle and saphenous AV-bundle as an “i*n vivo bioreactor*” to construct the pre-vascularized grafts successfully.

In this study, inferior epigastric AV-bundle were adopted based on the merits such as superficial anatomical location and adequate length. More importantly, it is considered to be a nonfunctional vessel. As engineered bone vascular network sources, inferior epigastric AV-bundle will not cause major damage to body functions and will meet the body’s need for bone tissue engineering. Furthermore, Tanaka Y et al. [10] demonstrated that flow-through type (distal un-ligated AV-bundle) enhanced angiogenesis but was weaker than ligation type (distal ligated AV-bundle). In the present study, the inferior epigastric AV-bundle was left un-ligated because of the increased risk of thrombosis of the ligation type which would affect the efficiency of the transportation nutrients and oxygen after anastomosis at the receptor site. In our experiment, the results demonstrated that abundant new capillaries were observed around the AV-bundle compared with the muscle encapsulated type.

In addition to the pre-existing vessel approach to increase the vascularization efficiency of engineered constructs, several techniques have supplemented angiogenic growth cytokines and cells. Undoubtedly, angiogenic cytokines such as VEGF and FGF could improve endothelial cell proliferation, cell migration, tube formation, and functional blood vessel formation both *in vitro* and *in vivo*[41-45]. However, because of their short biological half life, long-lasting and controlled-release delivery systems still remains a challenge. Cells that can secrete angiogenic cytokines could partially solve the problem. Bleiziffer O et al. [20] seeded endothelial progenitor cells (EPCs) into bioartificial matrices together with the AV-loop, demonstrating that EPCs may hold promise to modulate blood vessel formation. Rouwkema J et al. [21] reported that human umbilical vein endothelial cells (HUVECs) are able to form a three-dimensional prevascular network *in vitro* in a bone tissue engineering. Unfortunately, the limited sources of EPCs and HUVECs have hampered their wide applications, which lead us to find another suitable cell candidate for the prefabrication of vascularized grafts.

To date, stem cell based tissue engineering is focused on BMSCs. Despite the controversies, some scholars have described that there are no differences on multilineage differentiation capacities between BMSCs and ADSCs [46,47]. Recently, ADSCs have been successfully and reliably differentiated into an endothelial cell lineage [22,48,49]. ADSCs are easier to obtain than BMSCs, have lower donor-site morbidity, and are available in larger numbers. In addition, the isolation efficacy of ADSCs does not change with advanced age, gender, obesity, renal failure or vascular disease [50]. ADSCs provide a clear advantage as an alternative source for endothelial cells. Many scholars reported that endothelial cells in different origin (adipose tissue, bone marrow, umbilical vein, or peripheral blood) have the similar capacity to accelerate the process of angiogenesis in tissue engineered implants in the absence of exogenous angiogenesis growth factors [51-53].

Angiogenesis is modulated through complex molecular signals mediated by various cytokines involving extracellular matrix remodeling, endothelial cells proliferation and migration, capillary differentiation and anastomosis. VEGF and FGF are vital angiogenic cytokines based on their capacities of regulating the budding and growth of new vessels from existing vessels [54]. They are predominantly produced in tissues that acquire new capillary networks [55]. In the present study, the highly expressed of VEGF-C and FGF-2 were detected in Group A compared to the other 3 groups at each time point based on our *in vivo* western blot assay. This was contributed to autocrine VEGF and FGF-2 from endothelial cells. Increased expression of VEGF and FGF-2 may promote the physiologic function of the endothelial cells. As anticipated, luminal sprouting from the inferior epigastric vein was observed at a faster rate than the other three groups. Particularly in Group B, vessel diameter, vessel density, VEGF and FGF-2 protein expression were found to be slightly higher than group C, but no statistical difference was observed. This may be attributed to the deficient number of rADSCs and the absence of the endothelial differentiated conditions *in vivo*.

The study of cell-scaffold or growth factor-scaffold composites *in vitro* and induced tissue and organ regeneration *in vivo* have been advanced by the development and extensive use three-dimensional porous biomaterials scaffold. Microarchitectures such as porosity, macropore shape, macropore size, and interconnections are critical characteristics to improve the efficiency the scaffolds [56,57] Macropores and interconnections between nearby macropores act as two constitutive components of scaffolds, which strongly influence cell migration and tissue penetration [56-58]. As previously reported, macropore size larger than 400 μm is facilitated for the progression of not only osteogenesis but also angiogenesis and suitable for bone tissue engineering application [59]. Furthermore, interconnections with the size of larger than 100 μm can facilitate mineralized bone tissue ingrowth and 5–15 μm for fibrous tissue [59].

In the present study, we adopted nHA-PA 66 biomaterials to construct the scaffold. The nHA-PA 66 composite which compounds inorganic material nHA and organic polymer material PA 66 dramatically resembles natural bone in its composition, structure, and mechanical properties (elastic modulus, bending strength and compressive strength), which is responsible for its good biocompatibility, osteoconductivity, and bioactivity [24-26]. Furthermore, the porosity, macropore size and interconnection pore size were also in accordance with the preferred parameters for bone tissue engineering scaffold. Compared with sole HA biomaterials, partially biodegradation could be observed of the nHA-PA 66 scaffold at 4 weeks after implantation by the presence of organic biomaterials (PA 66), which may result in more newly formed tissue ingrowth [38].

In all groups, osteoid and osteoblast could not be observed based at 2 or 4 weeks after surgery. Our in *vivo* western blot confirmed that no BMP-2 protein was detected. The possible reasons may be that: (1) The nHA-PA 66 scaffold we adopted was not the osteoinductive biomaterials; (2) In group A, C and D, bone progenitor cells may not present in the operative areas, which are crucial for osteogenesis; (3) In group B, local environment at the operative region could not promote the osteogenic differeation of ADSCs. In the subsequently experiments, we focused on the co-cultured rADSCs-endo cells and osteoblasts together with porous scaffolds and the AV-bundle in order to prefabricate osseous construct with axial vessels simultaneously.

## Conclusion

In summary, compared with simply AV-bundle implantation and muscular wrapping method, the combination of pre-differentiated rADSCs (endothelial differentiation) and AV-bundle will achieve rapidly angiogenesis of biomaterial scaffold.

## Competing interests

The authors declare that they have no competing interests.

## Authors’ contributions

Conceived and designed the experiments: KW. Performed the experiments: PY and CW. Analyzed the data: XD. Contributed reagents/materials/analysis tools: XD and KW. Wrote the manuscript: PY, XH, ZD, HM and JS. All authors read and approved the final manuscript.

## Pre-publication history

The pre-publication history for this paper can be accessed here:

http://www.biomedcentral.com/1471-2474/14/318/prepub

## Supplementary Material

Additional file 1: Figure S1 Western blot results confirmed that there was no BMP-2 expression in all groups at 2 and 4 weeks. Click here for file
