# Supplementary figures and images for: Development of a new pre-vascularized tissue-engineered construct using pre-differentiated rADSCs, arteriovenous vascular bundle and porous nano-hydroxyapatide-polyamide 66 scaffold
Source: BMC Musculoskelet Disord. 2013 Nov 8;14:318. doi: 10.1186/1471-2474-14-318 (PMC3826526; doi:10.1186/1471-2474-14-318)

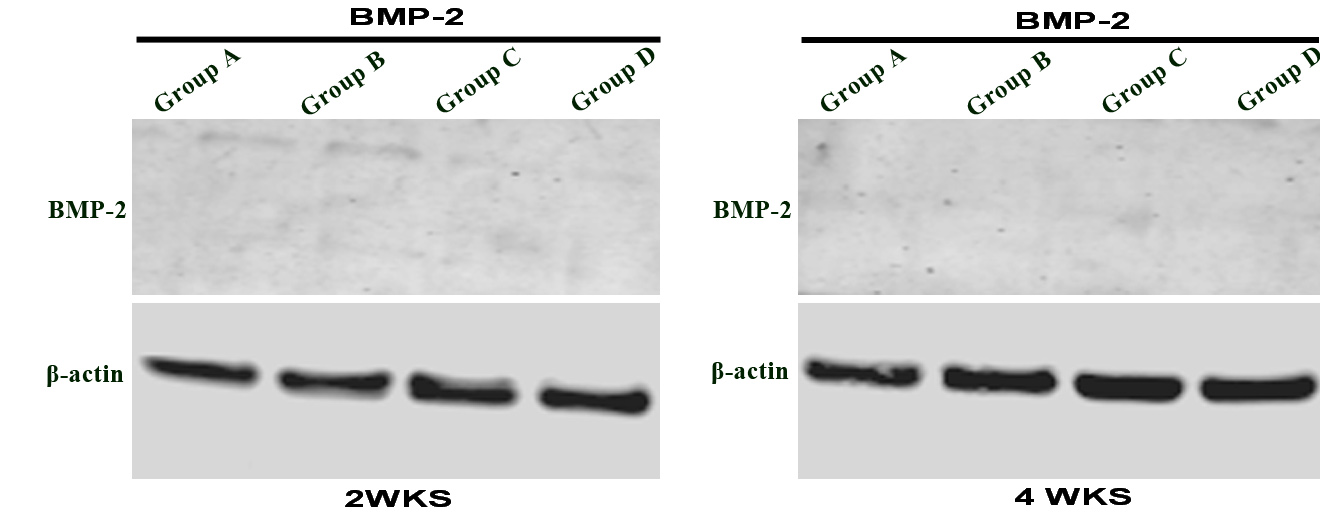

Supplement: Additional file 1: Figure S1 — Western blot results confirmed that there was no BMP-2 expression in all groups at 2 and 4 weeks. [file 1471-2474-14-318-S1.jpeg]
